# Respiratory Viral Detection in Children Hospitalized With Pneumonia During Periods of Major Population Disruptions in Nepal, 2014-2018

**DOI:** 10.1093/jpids/piaf052

**Published:** 2025-06-11

**Authors:** Shrijana Shrestha, Sanjeev M Bijukchhe, Brian Wahl, Michael J Carter, Rama Kandasamy, Meeru Gurung, Peter J O’Reilly, Marie Voice, Bhishma Pokhrel, Puja Amatya, Saugat Bhandari, Sonu Shrestha, Sarah Kelly, Dominic F Kelly, Stephen Thorson, David R Murdoch, Colin Fink, Maria Deloria Knoll, Andrew J Pollard

**Affiliations:** Patan Academy of Health Sciences, Patan Hospital, Satdobato Road, 44700 Lalitpur, Nepal; Patan Academy of Health Sciences, Patan Hospital, Satdobato Road, 44700 Lalitpur, Nepal; Oxford Vaccine Group, Department of Paediatrics, University of Oxford and the NIHR Oxford Biomedical Research Centre, Oxford, United Kingdom; Department of Epidemiology of Microbial Diseases, Yale School of Public Health, New Haven, CT, United States; International Vaccine Access Center, Department of International Health, Johns Hopkins Bloomberg School of Public Health, Baltimore, MD, United States; Centre for Human Genetics, University of Oxford, Oxford, United Kingdom; Paediatric Intensive Care Unit, Oxford University Hospitals NHS Foundation Trust, Oxford, United Kingdom; Faculty of Medicine and Health, University of Sydney, Sydney, Australia; National Centre for Immunisation Research and Surveillance, The Children’s Hospital at Westmead, Westmead, Australia; Patan Academy of Health Sciences, Patan Hospital, Satdobato Road, 44700 Lalitpur, Nepal; Oxford Vaccine Group, Department of Paediatrics, University of Oxford and the NIHR Oxford Biomedical Research Centre, Oxford, United Kingdom; Micropathology Ltd, University of Warwick, Coventry, United Kingdom; Patan Academy of Health Sciences, Patan Hospital, Satdobato Road, 44700 Lalitpur, Nepal; Patan Academy of Health Sciences, Patan Hospital, Satdobato Road, 44700 Lalitpur, Nepal; Patan Academy of Health Sciences, Patan Hospital, Satdobato Road, 44700 Lalitpur, Nepal; Oxford Vaccine Group, Department of Paediatrics, University of Oxford and the NIHR Oxford Biomedical Research Centre, Oxford, United Kingdom; Oxford Vaccine Group, Department of Paediatrics, University of Oxford and the NIHR Oxford Biomedical Research Centre, Oxford, United Kingdom; Oxford Vaccine Group, Department of Paediatrics, University of Oxford and the NIHR Oxford Biomedical Research Centre, Oxford, United Kingdom; Patan Academy of Health Sciences, Patan Hospital, Satdobato Road, 44700 Lalitpur, Nepal; Department of Pathology and Biomedical Science, University of Otago, Christchurch, New Zealand; Micropathology Ltd, University of Warwick, Coventry, United Kingdom; International Vaccine Access Center, Department of International Health, Johns Hopkins Bloomberg School of Public Health, Baltimore, MD, United States; Oxford Vaccine Group, Department of Paediatrics, University of Oxford and the NIHR Oxford Biomedical Research Centre, Oxford, United Kingdom

**Keywords:** respiratory viruses, pneumonia, pediatrics

## Abstract

**Background:**

Respiratory viruses commonly cause pneumonia in children. We aimed to identify respiratory viral nucleic acids in the nasopharynx of children admitted with pneumonia from 2014 to 2018, a period including a major earthquake (April 2015), pneumococcal conjugate vaccine (PCV10) introduction (August 2015), and a fuel shortage (October 2015 to March 2016).

**Methods:**

Children 2 months to 14 years admitted to Patan Hospital between March 2014 and February 2018 with a clinical diagnosis of pneumonia had nasopharyngeal swabs collected and tested with a multiplex panel for the presence of genetic material from 23 respiratory pathogens.

**Results:**

Of 1343 children with pneumonia, 974 (72.5%) had the nucleic acids of at least one respiratory virus in the nasopharynx. The median age of children with any viral genetic material detected was lower than those without (1.18, IQR: 0.59-2.39 years; vs 2.01 years, IQR: 0.81-4.34 years; *P* < .001). Commonly detected viral nucleic acids included those of respiratory syncytial virus (RSV) (21.0%), rhino/enterovirus (30.8%), and parainfluenza (7.4%). The odds of detecting any respiratory viral genetic material in children with pneumonia increased by 1.88 (95% confidence interval: 1.15, 3.06) in the year after the earthquake, when there were several aftershocks and a fuel crisis, relative to other periods and accounting for other potential confounding factors.

**Conclusions:**

These findings highlight the importance of viral diagnostics in pediatric pneumonia and suggest that public health measures addressing environmental conditions during disasters might help reduce respiratory infections.

## INTRODUCTION

Pneumonia is one of the most common causes of childhood hospital admissions and the major cause of morbidity and mortality in children in low- and middle-income countries (LMICs).^1,2^ With conjugate vaccines against *Haemophilus influenzae* type b (Hib) and *Streptococcus pneumoniae* (pneumococcus), improved immunization coverage against these bacterial pathogens, and greater use of diagnostic tests, viral pathogens have been more commonly reported in cases with pneumonia.^1^

Common respiratory viruses identified among pneumonia cases are respiratory syncytial virus (RSV), rhinovirus, influenza, parainfluenza, human metapneumovirus (hMPV), adenovirus, and parainfluenza virus.^1,3-5^ The Pneumonia Etiology Research for Child Health (PERCH) study conducted in 7 settings with high pneumonia burdens estimated that viruses accounted for 61.4% of hospitalized pneumonia cases in children <5 years, with RSV being the most commonly associated virus.^4^

The estimated incidence of pneumonia in Nepal in 2015 was 259 cases per 1000 children less than 5 years of age, higher than the LMIC average of 231 cases per 100 children.^2^ In Nepal, many health facilities lack blood culture services, and point-of-care viral diagnostic tests are not readily available at public hospitals. Hence, a clinical diagnosis, with or without chest X-ray and blood investigations, is the primary basis for determining the course of pneumonia treatment.

Several events affecting pneumonia epidemiology have occurred in Nepal since 2000. In April and May 2015, Nepal witnessed 2 large earthquakes (7.8 and 7.3 on the Richter scale), followed by several aftershocks for the next 3 months.^6^ The effects of the earthquakes included approximately 8000 acute deaths, substantial population migration, crowded housing, and increased air pollution.^7,8^ Following the earthquakes from October 2015 to March 2016, an informal blockade on the India–Nepal border led to an increase in the use of firewood as an alternative fuel for cooking and heating, which exacerbated air pollution.^9^ These population disruptions occurred in the context of new vaccines that were introduced for respiratory pathogens, namely *H influenzae* type b (Hib) vaccine in 2005 and the 10-valent pneumococcal conjugate vaccine (PCV10) in August 2015.

As part of a larger study assessing the impact of the introduction of PCV10 in Nepal,^10^ we aimed to determine the presence of genetic material associated with common circulating respiratory viruses in the nasopharynx (NP) of children hospitalized with a clinical diagnosis of pneumonia. We further sought to characterize viral nucleic acid-positive cases for demographic, clinical, and seasonal factors. We also assessed the impact of the earthquake and fuel crisis on the presence of respiratory viruses in the NP.

## MATERIALS AND METHODS

### Study Population

Children 2 months to 14 years admitted with a clinical diagnosis of pneumonia to Patan Hospital between March 20, 2014, and February 29, 2018, were recruited after written informed consent. Eligible children were identified by study staff from daily admission logs based on the attending clinician’s diagnosis of pneumonia. At Patan Hospital, children were admitted with a diagnosis of pneumonia if they presented with fever, cough, or fast breathing, along with one or more of the following: signs of respiratory distress (ie, use of accessory muscles, decreased SpO₂), abnormal chest examination findings (ie, decreased breath sounds and/or crepitations), severity indicators (ie, lethargy, vomiting, convulsions, inability to drink or breastfeed), or mortality risk factors (ie, comorbidities, failure to improve after oral antibiotics, referral from another hospital). An attending pediatrician verified each diagnosis during twice-daily ward rounds, deliberately excluding patients with reactive airway disease or uncomplicated upper respiratory viral infections. All children with pneumonia clinical diagnoses had chest radiographs interpreted using standardized criteria.^11^ Apart from PCV10, no other new vaccines, including the influenza vaccine, were introduced into Nepal’s national immunization program during the study period.

### Sample Collection and Processing

NP swabs were collected from all participants by trained research fellows following WHO Pneumococcal Carriage Working Group Guidelines.^12^ A single flocked swab (ThermoFisher Scientific, UK) was passed through a naris to the NP, rotated 180 degrees, and removed. The swab was immediately placed into skim milk, tryptone, glucose, and glycerine (STGG) media and transported to the microbiology laboratory onsite at Patan Hospital, Samples were frozen at −80 °C and shipped to the UK for further analysis. Viral RNA/DNA was detected using the NxTAG Respiratory Pathogen Panel (RPP) from Luminex, which targets RSV (A and B), influenza A (H1, H3), influenza B, parainfluenza types 1-4, rhino/enterovirus, human coronaviruses (229E, OC43, NL63, HKU1), hMPV, human bocavirus, and adenovirus.

### Statistical Analysis

Patient characteristics were described using standard descriptive statistics. The number and proportion of children with viral RNA/DNA detected are presented according to years and months of the year. Continuous variables were compared between groups using the Student’s *t*-test and the Mann–Whitney test, as appropriate. Categorical variables were assessed using Pearson’s chi-squared test. We used logistic regression to estimate odds ratios of detecting viral genetic material in the NP of children hospitalized with pneumonia for several factors controlling for possible confounding variables. Specifically, we included calendar month to account for seasonality, age in years, nutritional status, gender, receipt of at least one dose of PCV10, chest X-ray interpretation, and study period (ie, pre-disturbances from March 2014 to March 2015; the earthquakes from April 2015 to September 2015; the fuel crisis from October 2015 to March 2016; and post-disturbances from April 2015 to February 2018). We considered *P*-values < .05 to be statistically significant. StataMP 18 was used for all the analyses.

### Ethics Statement

The study was conducted in accordance with the guidance of the International Conference on Harmonisation-Good Clinical Practice (ICH-GCP) and was approved by the Oxford Tropical Research Ethics Committee (OXTREC reference, 05-14) and the Nepal Health Research Council (NHRC reference, 04/2014). Participant information leaflets were provided in Nepali to the parents/guardians and to the child if appropriate. Informed written consent was taken, and participants were free to withdraw from the study at any point.

## RESULTS

**Figure 1.**
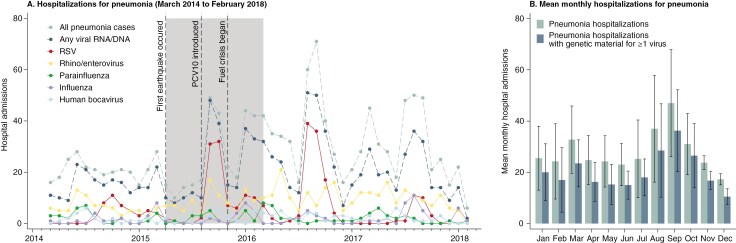
Timeline of Clinical Cases With a Clinical Diagnosis of Pneumonia and Viral Pathogens With Genetic Material Between March 2014 and February 2018. (A) Shows the number of hospital admissions for all cases with clinically diagnosed pneumonia (dashed line), cases of clinically diagnosed pneumonia in which any viral genetic material was identified (dashed line), and individual viruses for which genetic material was identified (solid lines). Viruses not shown include human metapneumovirus, adenovirus, and human coronavirus. The shaded area shows the timing of 3 important events in Nepal: the first of 2 major earthquakes, the introduction of PCV10 in the national immunization program, and the fuel crisis. Hospital admissions can have more than one virus for which genetic material was identified, so monthly totals for individual viruses could exceed the cases with pneumonia in which a virus was identified. (B) Shows the mean monthly hospitalizations for pneumonia by month with 95% confidence intervals for all hospitalizations for pneumonia in teal, and hospitalizations for pneumonia for which at least one viral respiratory pathogen was identified.

Between March 20, 2014, and February 29, 2018, 1343 children were enrolled and had NP results (Figure 1A). Of these, 63.1% were <2 years and 60.5% were male (Table 1). September had the highest average number of admitted clinical cases with pneumonia (Figure 1B). Most (974/1343; 72.5%) had viral nucleic acids associated with one or more viruses detected in the NP. *Streptococcus pneumoniae* was detected in 35.7% (480/1343) children, of which 34.2% (164/480) were PCV10-type serotypes and 10.8% (51/480) were additional PCV13-type serotypes. *Mycoplasma pneumoniae* PCR was positive in 1.6% of children (21/1343) and *Chlamydia pneumoniae* in 0.5% of children (6/1343). No children with pneumonia were positive for *Legionella pneumophila*.

**Table 1.** Profile of Clinical Cases With Clinically Diagnosed Pneumonia Admitted Between March 2014 and February 2018

	2014, from March	2015	2016	2017	2018, through February	Total	*P*-value
Total cases of clinically diagnosed pneumonia (%)	210 (15.6)	267 (19.9)	459 (34.2)	379 (28.2)	28 (2.1)	1343 (100.0)	-
Age group (%)							.1861
<6 months	42 (20.0)	55 (20.6)	91 (19.8)	55 (14.5)	10 (35.7)	253 (18.8)	
6-12 months	42 (20.0)	59 (22.1)	106 (23.1)	78 (20.6)	3 (10.7)	288 (21.4)	
1-2 years	51 (24.3)	57 (21.4)	108 (23.5)	86 (22.7)	5 (17.9)	307 (22.9)	
2-5 years	47 (22.4)	60 (22.5)	107 (23.3)	108 (28.5)	9 (32.1)	331 (24.7)	
5-14 years	28 (13.3)	36 (13.5)	47 (10.2)	52 (13.7)	1 (3.6)	164 (12.2)	
Gender (%)							.2723
Male	133 (63.3)	172 (64.4)	276 (60.1)	218 (57.5)	14 (50.0)	813 (60.5)	
Female	77 (36.7)	95 (35.6)	183 (39.9)	161 (42.5)	14 (50.0)	530 (39.5)	
Chest X-ray findings							.0001
Normal	69 (35.8)	123 (48.4)	250 (55.0)	208 (56.7)	21 (75.0)	671 (51.7)	
PEP	84 (43.5)	87 (34.2)	155 (34.1)	110 (30.0)	5 (17.9)	441 (34.0)	
Infiltrates	36 (18.7)	41 (16.1)	48 (10.6)	46 (12.5)	2 (7.1)	173 (13.3)	
Unknown	4 (2.1)	3 (1.2)	2 (0.4)	3 (0.8)	0 (0.0)	12 (0.9)	
Presence of respiratory virus RNA/DNA in NP swab							<.0001
No virus	59 (28.1)	51 (19.1)	115 (25.1)	132 (34.8)	12 (42.9)	369 (27.5)	
1 virus	118 (56.2)	162 (60.7)	262 (57.1)	202 (53.3)	15 (53.6)	759 (56.5)	
> 1 virus	33 (15.7)	54 (20.2)	82 (17.9)	45 (11.9)	1 (3.6)	215 (16.0)	
Presence of pneumococcus in NP swab^*^							.0453
No pneumococcus	124 (59.1)	174 (65.1)	312 (68.0)	239 (63.1)	14 (50.0)	863 (64.3)	
Non-PCV types	42 (20.0)	48 (18.0)	81 (17.7)	82 (21.6)	12 (42.9)	265 (19.7)	
PCV10 types	35 (16.7)	37 (13.9)	50 (10.9)	40 (10.6)	2 (7.1)	164 (12.2)	
Additional PCV13 types	9 (4.3)	8 (3.0)	16 (3.5)	18 (4.8)	0 (0.0)	51 (3.8)	

Abbreviations: NP = nasopharynx; PEP = primary endpoint pneumonia; PCV = pneumococcal conjugate vaccine.

Children admitted to Patan Hospital between March 2014 and February 2018 with a clinical diagnosis of pneumonia had their nasopharynx swabbed to detect the presence of respiratory viral pathogens and bacteria (ie, *Streptococcus pneumonia*, *Mycoplasma pneumoniae*, *Chlamydia pneumoniae*, and *Legionella pneumophila*).

^*^46.6% of all cases with pneumonia reported recent antibiotic exposure.

Table 2 provides details on the detection of viral RNA/DNA in the NP of patients with pneumonia. Rhino/enterovirus nucleic acids were the most commonly detected (414/1343, 30.8%), followed by RSV (282/1343; 21.0%). All other viral nucleic acids were detected on average 6.0%-8.0%, except human coronaviruses (pre-SARS-CoV-2), the least commonly detected (36/1343, 2.7%). RSV A accounted for 74.5% (210/282) of all cases with pneumonia with any RSV subtype in the NP. Influenza A accounted for 75.0% (72/96) of all influenza subtypes for which RNA was detected. Parainfluenza 3 and 1 contributed 81.8% (81/99) of all parainfluenza subtype RNA detected. Coronavirus OC43 was the most common coronavirus variant for which RNA was detected (*n* = 19; 50.0%).

**Table 2.** Profile of Cases With Clinically Diagnosed Pneumonia With Viruses Detected in the NP

	Total	Any viru		RSV	Rhino/enterovirus	Parainflu-enza	Influenza	Human bocavirus	hMPV	Adenovirus	Human coronavirus
		Yes	No	Yes	Yes	Yes	Yes	Yes	Yes	Yes	Yes
Total (%)	1343	974 (72.5)	369 (27.5)	282 (21.0)	414 (30.8)	99 (7.4)	96 (7.1)	94 (7.0)	89 (6.6)	76 (5.7)	36 (2.7)
Age group (%)											
<6 months	253 (18.8)	198 (20.3)	55 (14.9)	97 (34.4)	72 (17.4)	18 (18.2)	9 (9.4)	9 (9.6)	21 (23.6)	4 (5.3)	9 (25.0)
6-12 months	288 (21.4)	231 (23.7)	57 (15.4)	79 (28.0)	93 (22.5)	25 (25.3)	23 (24.0)	25 (26.6)	20 (22.5)	23 (30.3)	7 (19.4)
1-2 years	307 (22.6)	236 (24.2)	71 (19.2)	63 (22.3)	89 (21.5)	31 (31.3)	16 (16.7)	26 (27.7)	28 (31.5)	20 (26.3)	7 (19.4)
2-5 years	331 (24.6)	230 (23.6)	101 (27.4)	41 (14.5)	106 (25.6)	22 (22.2)	36 (37.5)	27 (28.7)	17 (19.1)	23 (30.3)	9 (25.0)
5-14 years	164 (12.2)	79 (8.1)	85 (23.0)	2 (0.7)	54 (13.0)	3 (3.0)	12 (12.5)	7 (7.4)	3 (3.4)	6 (7.9)	4 (11.1)
*P*-value		<.001		<.001	.74	.02	.008	.051	.02	.009	.90
Male (%)	813 (60.5)	584 (60.0)	229 (62.1)	170 (60.3)	254 (61.4)	61 (61.6)	55 (57.3)	63 (67.0)	48 (53.9)	42 (55.3)	19 (52.8)
*P*-value		0.48		0.92	0.68	0.82	0.50	0.18	0.19	0.33	0.33
Chest X-ray findings (%)											
Normal	671 (50.0)	536 (56.8)	135 (38.2)	172 (63.2)	209 (51.7)	54 (56.3)	60 (66.7)	47 (54.0)	53 (60.2)	43 (59.7)	13 (38.2)
PEP	441 (32.8)	270 (28.6)	171 (48.4)	48 (17.6)	141 (34.9)	23 (24.0)	18 (20.0)	28 (32.2)	25 (28.4)	21 (29.2)	18 (52.9)
Infiltrates	173 (12.9)	131 (13.9)	42 (11.9)	50 (18.4)	52 (12.9)	19 (19.8)	11 (12.2)	12 (13.8)	9 (10.2)	8 (11.1)	3 (8.8)
Unknown	12 (0.9)	7 (0.7)	5 (1.4)	2 (0.7)	2 (0.5)	0 (0)	1 (1.1)	0 (0.0)	1 (1.1)	0 (0.0)	0 (0.0)
*P*-value		<.001		<.001	.70	.053	.02	.79	.40	.48	.12
Quarter (%)											
1st	330 (24.6)	242 (24.8)	88 (23.8)	40 (14.2)	88 (21.3)	30 (30.3)	34 (35.4)	18 (19.1)	38 (42.7)	21 (27.6)	20 (55.6)
2nd	288 (21.4)	186 (19.1)	102 (27.6)	7 (2.5)	119 (28.7)	34 (34.3)	7 (7.3)	29 (30.9)	5 (5.6)	14 (18.4)	6 (16.7)
3rd	437 (32.5)	331 (34.0)	106 (28.7)	143 (50.7)	111 (26.8)	27 (27.3)	41 (42.7)	25 (26.6)	22 (24.7)	26 (34.2)	6 (16.7)
4th	288 (21.4)	215 (22.1)	73 (19.8)	92 (32.6)	96 (23.2)	8 (8.1)	14 (14.6)	22 (23.4)	24 (27.0)	15 (19.7)	4 (11.1)
*P*-value		.007		<.001	<.001	<.001	<.001	.08	<.001	.84	<.001
Genetic material for >1 viral pathogen detected (%)	215 (16.0)	215 (16.0)	0 (0.0)	81 (28.7)	143 (34.5)	35 (35.4)	17 (17.7)	69 (73.4)	26 (29.2)	48 (63.2)	20 (55.6)
*P*-value		-		<.001	<.001	<.001	.64	<.001	<.001	<.001	<.001
Pneumococcus detected in NP (%)	480 (35.7)	340 (34.9)	140 (37.9)	82 (29.1)	162 (39.1)	40 (40.4)	35 (36.5)	31 (33.0)	32 (36.0)	25 (32.9)	19 (52.8)
*P*-value		.30		.009	.08	.31	.88	.56	.97	.59	.03

*P*-values are derived from chi-squared tests comparing cases with and without the indicated virus isolated from the NP. RSV includes RSV A and B. Parainfluenza includes parainfluenza 1-4. Influenza includes influenza A and B. Human coronavirus includes 229E, OC43, NL63, or HKU1. See Supplementary Table 1 for results for cases without the indicated virus.

**Figure 2.**
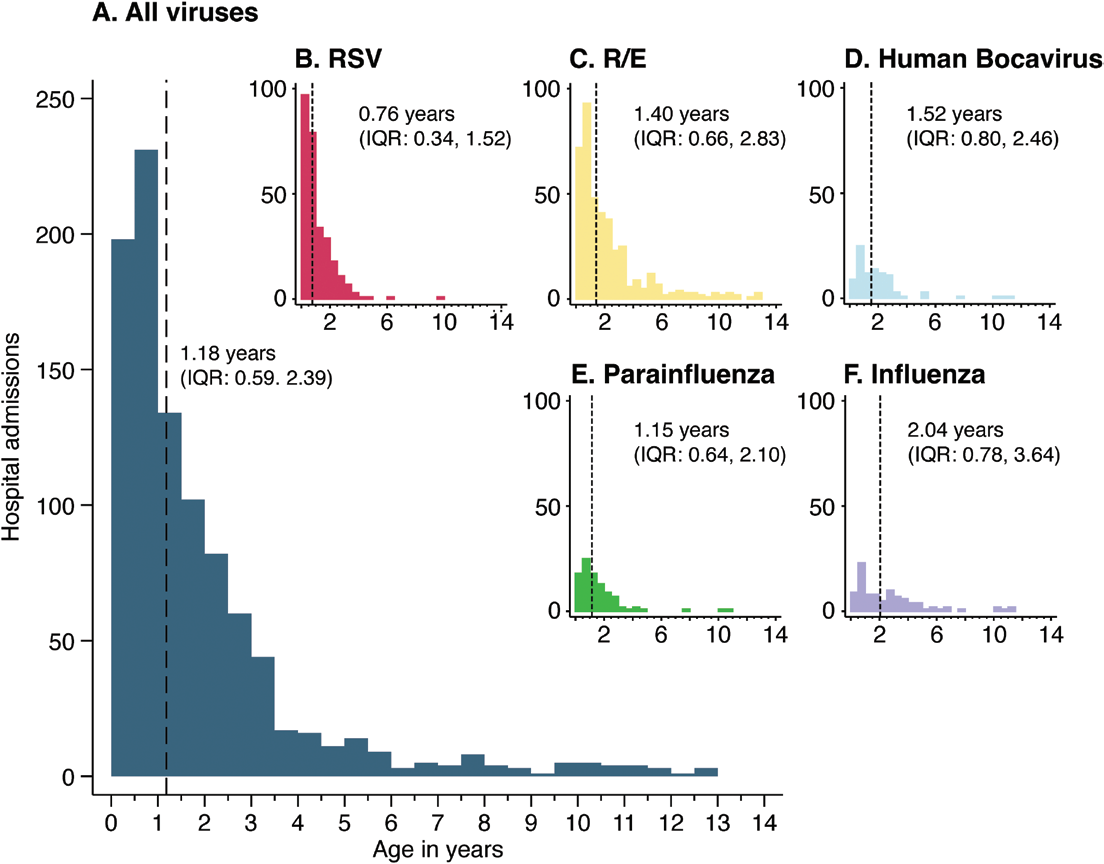
Age Distribution of Viruses in the Nasopharynx of Cases of Clinical Pneumonia for Which Genetic Material Was Identified. (A) Shows the age distribution, the median age with the dashed line and in the text, and the IQR for age in the text for clinical cases with pneumonia with genetic material for at least one virus isolated from the NP; (B-F) shows the same for RSV, R/E, human bocavirus, parainfluenza, and influenza. IQR = interquartile range; RSV = respiratory syncytial virus; R/E = rhino/enterovirus.

The profile of patients with pneumonia with viral genetic material detected in the NP, including age distribution, is also described in Table 2 and Figure 2. Children with a positive PCR for any respiratory virus were younger than children without a virus detected (median: 1.18 years vs 2.01 years; *P* < .001). Children who tested positive for RSV RNA were younger (median: 0.76 years) than those positive for other respiratory virus genetic material (median: 1.44 years; *P* ≤ .001).

Five prominent peaks in hospitalizations for pneumonia occurred during the study period, all after April 2015, when the first major earthquake occurred (Figure 1A). Two of the 5 peaks of hospitalizations for pneumonia appeared to have been driven by RSV and rhino/enterovirus together. Genetic material associated with these pathogens was found in 74.3% and 66.7% of all hospitalizations for pneumonia from August to October 2015 and 2016, respectively. The 2015 peak corresponded with the post-earthquake period. From January to May 2016, we observed an early and prolonged secondary peak of hospitalizations for pneumonia. While 77.2% of hospitalizations for pneumonia had a viral RNA/DNA isolated from the NP during this time, no single pathogen appears responsible for this peak; rhino/enterovirus genetic material was isolated from 32.5% of patients with pneumonia in the first 3 months of 2016—the highest single pathogen isolated during this time. The first 3 months of this period corresponded with the fuel crisis, during which biomass fuels were the primary source of cooking and heating for many households.

The seasonality of all hospitalizations for pneumonia and those in which viral nucleic acids were detected were highly correlated throughout the study period (Figure 1A and B). Approximately 2 peaks occurred within each year. A primary peak of pneumonia occurred approximately from August to September, during the late monsoon and early autumn, and a secondary peak occurred approximately from January to March, during the late winter and early spring (Figure 1B). The monthly variance in hospitalizations for pneumonia was 7.83 times greater after April 2015, when the first earthquake occurred, compared to the period before the earthquake (*P* < .001).

**Figure 3.**
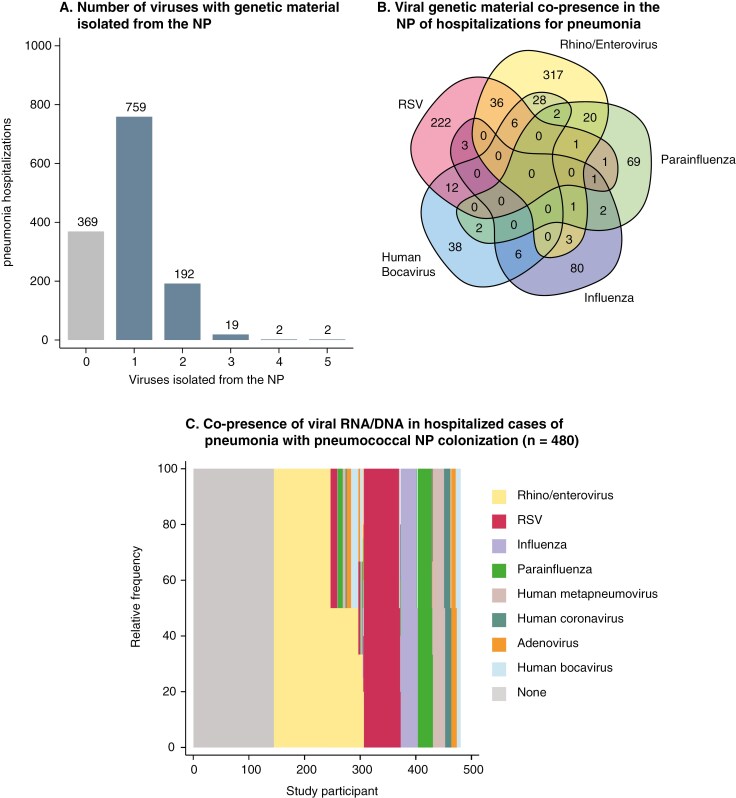
Co-Presence of Viral Respiratory Pathogen Nucleic Acids in Cases of Clinically Diagnosed Pneumonia. (A) Shows the distribution of viral genetic material present in the NP of clinical hospitalizations for pneumonia. The numbers above the bars are the absolute number of hospitalizations for each group. (B) Shows the co-presence of viral RNA/DNA in the NP for the 5 most prominent viruses isolated from children hospitalized with clinical pneumonia. (C) Shows the co-presence of viral genetic material in the NP among all 480 children with pneumococcal NP colonization. Each study participant with pneumococcal NP colonization is represented as a bar on the *x*-axis. 140 cases with pneumonia with pneumococcal NP colonization had 0 viruses isolated from their NP, 265 had one virus, 63 had 2 viruses, and 12 had 3 or more viruses. Study participants are sorted based on the frequency of viral pathogens identified. NP = nasopharynx, RSV = respiratory syncytial virus.

Of all enrolled children, 17.1% (229/1343) did not have a viral RNA/DNA nor a bacterium isolated from the NP, although 46.4% reported receiving an antibiotic recently. Genetic material for more than one virus was isolated from the NP in 16.0% (215/1343) of cases (Figure 3A). The most common combinations of viral nucleic acids were RSV-rhino/enterovirus (3.70%; 36/974) and RSV-rhino/enterovirus-human bocavirus (2.90%; 28/974)—Figure 3B. Pair-wise adjusted odds ratios (aORs) for detecting specific viral genetic material and bacterial pathogens among those with other pathogens are described in Table 3.

**Table 3.** Adjusted Odds Ratio of Detecting Bacterial and Viral Pathogen Genetic Material in Cases With Clinical Diagnosis of Pneumonia With Other Bacterial and Viral Pathogens

					Independent variable										
					Viruses								Bacteria		
			Number positive	Prevalence	RSV	Rhinovirus/Enterovirus	Parainfluenza	Influenza	Human bocavirus	hMPV	Adenovirus	Human coronavirus	*Streptococcus pneumoniae*	*Mycoplasma pneumoniae*	*Chlamydia pneumoniae*
**Dependent variable**	Viruses	RSV	282	21.0		0.31	0.10	0.10	1.28	0.05	0.93	0.31	0.70	1.31	-
		Rhinovirus enterovirus	414	30.8			0.61	0.08	1.15	0.56	0.91	0.75	1.17	0.12	1.85
		Parainfluenza	99	7.4				0.42	0.46	0.27	0.39	2.14	1.09	0.93	-
		Influenza	96	7.1					0.97	-	0.32	0.52	1.00	0.73	-
		Human bocavirus	94	7.0						0.57	3.06	-	0.74	0.81	-
		hMPV	89	6.6							0.34	2.10	0.96	0.59	-
		Adenovirus	76	5.7								0.51	0.71	-	-
		Human coronavirus	36	2.7									1.92	2.87	-
	Bacteria	*Streptococcus pneumoniae*	480	35.7										0.32	0.54
		*Mycoplasma pneumoniae*	21	1.6											-
		*Chlamydia pneumoniae*	6	0.4											

Abbreviations: hMPV = human metapneumovirus; RSV = respiratory syncytial virus.

Cells indicate the adjusted odds ratios of detecting a viral RNA/DNA or bacterial pathogen (left axis), comparing those with other viral or bacterial pathogens (top axis). Adjusted odds ratios also account for period, gender, age, nutritional status, chest X-ray results, and whether the child received one or more doses of PCV10. Blue cells indicate statistically significant adjusted odds ratios less than 1, and orange cells indicate statistically significant adjusted odds ratios greater than 1.

Among cases with pneumonia with pneumococcus detected in the NP, viral RNA/DNA were isolated from 70.8% (340/480), which was similar to the prevalence of viral nucleic acids in those without pneumococcal colonization (73.5%; 634/863; *P* = .301). The most common viral genetic material present—either alone or with others—were rhino/enterovirus (33.8%; 162/480), RSV (17.1%; 82/480), and influenza (7.3%; 35/480)—Figure 3C. Viral genetic material was isolated from 100.0% (21/21) of cases with pneumonia with *M pneumoniae* detected in the NP and 100.0% (6/6) of cases with *C pneumoniae* in the NP.

The results of chest X-rays for all cases with pneumonia are included in Table 2. Among all children with pneumonia, endpoint consolidation was identified in 34.0% (441/1297) children, infiltrates only were identified in 13.3% (173/1297), 51.7% (671/1297) were normal, and 0.9% (12/1297) were uninterpretable. Among pneumonia cases with any viral nucleic acids detected, 944 had chest X-rays available for interpretation—28.6% (270/944) were reported as endpoint consolidation (PEP), 13.9% (131/944) as infiltrates only, 56.8% (536/944) as normal, and 0.7% (7/944) as uninterpretable. Chest X-ray results significantly differed when stratified by whether viral RNA/DNA was identified (*P* < .001). As 205 children with chest X-rays available for interpretation had genetic material associated with more than one virus detected, there could be some overlap in the radiographic findings for each pathogen.

The median hospital stay among all the children with clinical pneumonia was 5 days (IQR: 3-7 days). The median hospital stay among children positive for any virus (5 days; IQR: 3, 7) was similar to those without a virus detected (6 days; IQR: 3, 9; *P* = .06). Pairwise comparisons of median hospital stay by virus are included in Supplementary Material; however, none of the pairwise comparisons were statistically significantly different.

Table 4 includes the results of a multiple logistic regression model. The aOR of isolating genetic material for one or more viruses from the NP of cases with pneumonia immediately following the earthquake and during the fuel crisis compared to before these events was 1.88 (95% CI: 1.15, 3.06). The aOR of identifying genetic material for a virus among children who received at least one dose of PCV10 compared to no doses was 0.63 (95% CI: 0.42, 0.94). Viral RNA/DNA was more common in younger children—the aOR for each additional year of life was 0.79 (95% CI: 0.70, 0.90). The aOR for isolating a viral nucleic acids from the NP comparing those with PEP to those with a normal chest X-ray was 0.49 (95% CI: 0.35, 0.68).

**Table 4.** Characteristics Associated With Detecting a Viral Pathogen Genetic Material in the NP of Children Hospitalized With Clinical Pneumonia

	Unadjusted odds ratios (95% confidence interval)	Adjusted odds ratios (95% confidence interval)
Period		
Pre-disturbances	REF	REF
Earthquake and fuel crisis	1.54 (1.05, 2.27)	1.88 (1.15, 3.07)
Post-disturbances	0.79 (0.58, 1.07)	0.87 (0.54, 1.40)
Gender		
Female	REF	REF
Male	0.92 (0.72, 1.17)	0.86 (0.64, 1.15)
Age (in years)	0.85 (0.81, 0.88)	0.76 (0.62, 0.94)
Nutritional status		
Normal	REF	REF
Moderate undernutrition	0.97 (0.70, 1.34)	1.12 (0.78, 1.60)
Severe undernutrition	0.76 (0.51, 1.14)	0.83 (0.54, 1.29)
Very severe undernutrition	0.64 (0.36, 1.12)	0.72 (0.39, 1.32)
Chest X-ray result		
Normal	REF	REF
PEP	0.40 (0.30, 0.52)	0.49 (0.35, 0.69)
Infiltrates only	0.79 (0.53, 1.17)	0.74 (0.48, 1.15)
Unknown	0.35 (0.11, 1.13)	0.39 (0.12, 1.32)
Receipt of PCV10 among children <2 years		
No PCV10	REF	REF
PCV10	0.63 (0.45, 0.88)	0.61 (0.39, 0.97)

Abbreviations: PEP = primary endpoint pneumonia; PCV10 = 10-serotype containing pneumococcal conjugate vaccine; REF = reference group. Logistic regression was used to calculate odds ratios and 95% confidence intervals. Adjusted logistic regression models included all covariates shown plus an indicator variable for month to account for seasonality. The coefficient for PCV10 was limited to those <2 years through an interaction term to account for age eligibility.

## DISCUSSION

In our study of children with clinically diagnosed pneumonia, conducted before the COVID-19 pandemic, 72.5% of children with clinical pneumonia had genetic material associated with one or more viruses detected in their NP. Notably, viral nucleic acid detection was highest following major earthquakes and during the period of acute fuel shortage.

These environmental disruptions substantially likely impacted respiratory infections through multiple pathways. The earthquakes led to population displacement and crowded temporary housing—a risk factor for respiratory infections^13,14^—and altered care-seeking behaviors as families relocated or faced transportation challenges. Many households used biomass fuels during the fuel crisis, contributing to poor indoor and ambient air pollution. Several epidemiological studies have shown associations between air pollution, respiratory conditions, and viral infections,^15^ with evidence suggesting air pollutants increase susceptibility to viral infections through inflammatory responses and immune system modulation.^16-18^ Our study demonstrated these effects through increased viral genetic material detection and pneumonia admissions during heightened air pollution from earthquake reconstruction and biomass fuel use. These findings suggest that targeted public health measures during disasters—such as providing clean fuel alternatives, improving temporary housing conditions, and implementing dust control—could help reduce respiratory infections during recovery periods.

Beyond the effects of these disruptions, the proportion of viral nucleic acids detected in our study was higher than reported in both the PERCH study (61.4%) and a recent meta-analysis of community-acquired pneumonia (55.0%).^4,5^ However, direct comparisons are limited by differences in case definitions, age ranges, and study designs. When we limit our analysis to children <5 years—the same as the PERCH study and the meta-analysis—75.9% of cases with pneumonia had genetic material for at least one virus detected in their NP. Previous studies from Nepal have shown varying levels of viral detection, with higher proportions in hospitalized children with severe pneumonia (2006-2008) and lower detection in community settings (2004-2007).^19,20^ Despite these differences in the detection of viral nucleic acids, the clinical pneumonia case definition we used in our study is aligned with the WHO criteria for the diagnosis of pneumonia in children less than 5 years of age.^21^

Seasonal differences have been reported in the distribution of viruses causing pneumonia,^17^ particularly for RSV. In our study, RSV showed clear seasonal peaks from August to October (late monsoon/early autumn), aligning with previously reported RSV patterns in Nepal that correlate with relative humidity.^20^ The virus likely contributed substantially to pneumonia hospitalizations, especially in 2015-2016. While RSV seasonality varies globally—March to June in the Southern Hemisphere and September to December in the Northern Hemisphere^22^—Nepal consistently demonstrates peaks during the monsoon season and winter/early spring.^20^ Viral infections may predispose individuals to bacterial pneumonia,^23,24^ with carriage of *S pneumoniae, H influenzae*, and *Moraxella catarrhalis* more common in RSV-infected children than in healthy controls.^25,26^ These clear seasonal patterns, RSV’s substantial contribution to hospitalizations, and interaction with other pathogens, highlight the potential benefits of RSV prevention through maternal vaccination or infant immunization.^27^

We observed a 36.7% relative reduction in NP viral detection among cases with pneumonia who received at least one dose of PCV10. Evidence suggests a broader role of PCVs in reducing lower respiratory tract infections, including viral-associated diseases^28,29^ and severe RSV infections in children.^30^ However, the observed effect size from our study warrants further investigation.

This study has important limitations. The lack of a control group limits our ability to infer the causal relationship between viral presence and pneumonia. We could not disaggregate rhinoviruses and enteroviruses, and they were, therefore, reported together. The study population may not represent the broader community as it was done only among hospitalized children with pneumonia in a tertiary care hospital. In addition, the earthquakes likely affected population movement and care-seeking behaviors, potentially changing the population that presented to the study hospital over time. This study was nested within a more extensive prospective study that planned to measure the impact of PCV10 on several endpoints.^10^ The COVID-19 pandemic affected the timeline for the parent study, and the planned 5-year follow-up was curtailed by 1 year. Therefore, we are unable to provide data after 2020. Other studies have found that the COVID-19 pandemic altered the circulation patterns of respiratory viruses, such as influenza and RSV, and may have influenced the overall burden and clinical presentation of viral pneumonia during this period by reducing co-infections through non-pharmaceutical interventions.^31^ Despite these constraints, our findings provide valuable insights into the epidemiology of respiratory viruses among children hospitalized with pneumonia in Nepal during a period of substantial events of the earthquakes and fuel crisis.

## CONCLUSIONS

Our study shows the changing epidemiology of clinical pneumonia in Nepal, particularly in the context of several events, including the introduction of PCV10 and substantial environmental disturbances. Standardizing tests for viral pathogens may help better understand pneumonia etiology and local viral epidemiology. This may be cost-effective in appropriately managing childhood pneumonia and supporting novel vaccine implementation in these settings.^32^ These findings emphasize the importance of incorporating viral diagnostics into routine pneumonia care, and the seasonal peaks of pneumonia associated with viruses highlighted in this study may assist with planning resource allocation across health services throughout the year. The reduction in viral detection among children who received PCV10 suggests broader protective effects of pneumococcal vaccination and the role it could play in mitigating viral-associated pneumonia. Public health interventions that focus on reducing air pollution and improving living conditions, especially following public health crises, could help decrease the incidence of respiratory viral infections. Understanding these dynamics is essential for developing effective strategies to combat childhood pneumonia and enhance public health resilience.

## Supplementary Material

piaf052_suppl_Supplementary_Materials

piaf052_suppl_Supplementary_PneumoNepal_Study_Members
