# Supplementary material for: Respiratory Viral Detection in Children Hospitalized With Pneumonia During Periods of Major Population Disruptions in Nepal, 2014-2018
Source: J Pediatric Infect Dis Soc. 2025 Jun 11;14(6):piaf052. doi: 10.1093/jpids/piaf052 (PMC12235135; doi:10.1093/jpids/piaf052)
Supplement: piaf052_suppl_Supplementary_PneumoNepal_Study_Members [file piaf052_suppl_supplementary_pneumonepal_study_members.docx]

**PneumoNepal Study Group members**

Imran Ansari, Krishna Govinda Prajapati, Rasmila Deshar, Tshering Yanyzon Gurung, Jyoti Kumari Yadav, Anshu Mallik, Kalpana Acharya, Subash Shrestha, Sonu Kumar Yadav, Diksha Nepal, Anushiya Kattel, Kushal Gautam, Roshan Jha, Shriya Bista, Swati Dhungel, Ruby Basi, Mamata Maharjan, Manisha KC, Jita Nepal, Laxmi Lama, Sabitri Bista, Arjun Kumar Budha, Shirty Raj Karnikar, Pallavi Gurung, Baikuntha Acharya, Saraswati Budhathok, Rupa Pudasaini, Tham Kumari Pun Magar (Patan Academy of Health Sciences, Kathmandu, Nepal); Sally Felle (Oxford Vaccine Group, University of Oxford, Oxford, UK); Katie Gorham, Rose Weeks, Alexandra Michel (International Vaccine Access Center, Johns Hopkins Bloomberg School of Public Health, Baltimore, MD, USA); Kate Park, Matt Smedley (Oxford Children's Hospital, Oxford University Hospitals NHS Foundation Trust, Oxford, UK).
